# Assessing mental health in individuals near thermal power plants and development of depression predictive model

**DOI:** 10.1038/s44184-025-00145-7

**Published:** 2025-10-28

**Authors:** Khaiwal Ravindra, Abhishek Kumar, Nitasha Vig, Suman Mor

**Affiliations:** 1 Department of Community Medicine & School of Public Health, Post Graduate Institute of Medical Education and Research (PGIMER), Chandigarh, 160012 India; 2 Department of Environment Studies, Panjab University, Chandigarh, 160014 India

**Keywords:** Psychology, Environmental sciences, Risk factors, Energy and society

## Abstract

Depression, anxiety, and stress are major mental health concerns globally, especially in India. This study examines the prevalence of mental health symptoms in overweight and normal BMI individuals living near thermal power plants and develops a depression prediction model using binary logistic regression using the DASS-21 score. A community-based cross-sectional study was conducted from October 2018 to March 2019, with data collected through face-to-face interviews. Socio-demographic factors like age, gender, cooking fuel type, and income were analyzed. Significant associations were found between stress and household air pollution (*p* = 0.011, OR = 17.408, 95% CI) and between anxiety and income below 1 lakh in normal BMI individuals (*p* = 0.045, OR = 0.303, 95% CI). Depression, anxiety, and stress were more prevalent in females. The depression prediction model demonstrated high performance with an ROC–AUC of 0.8754. These findings highlight the need to address environmental and socio-demographic factors to protect mental health in populations living near thermal power plants.

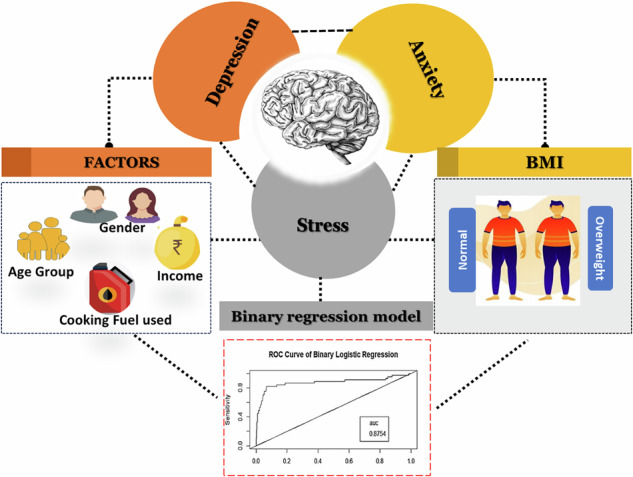

## Introduction

Mental health disorders are a leading cause of global disability with the World Health Organization (WHO) identifying them as a significant burden, especially among individuals aged 15–44, where they account for three out of the top ten causes of disability^1,2^. In India, the National Mental Health Survey reported a lifetime prevalence of common mental disorders at 12.3%^3^. Depression, anxiety, and stress are key indicators of mental health, and the failure to address these conditions can lead to severe individual and societal consequences^4^.

Depression, as defined by the American Psychiatric Association, is characterized by feelings of stress and loss of interest, leading to emotional and physical impairments lasting at least 2 weeks^5^. Stress, typically a short-term response to external factors, causes tension in the body or mind^6^. Anxiety, which shares symptoms with depression such as sleep disturbances, fatigue, irritability, is more persistent and can occur without any apparent trigger^7^. In 2015, depression affected 322 million people globally, contributing to years lived with disability. At the country level, India accounted for 18% of the global burden and an estimated 57 million cases^8^.

These findings highlight the significant impact of depression on individuals and communities worldwide, underscoring the need for effective prevention and management strategies to address the mental health issue. In India, coal- thermal power plants are the primary source of electricity^9^ and produce large amounts of fly ash, a by-product of coal combustion that contains hazardous metals at concentrations 2–10 times higher than in the original coal^10^. These plants are often located in low-income areas and disproportionately affect marginalized populations.

Although research has shown that exposure to fly ash negatively impacts neurodevelopment and mental health^11^, there are limited studies into the specific effects of coal ash exposure on mental health issues among individuals living near coal thermal power plants, nor their association with factors such as age, gender, income, and body mass index (BMI). For example, studies have shown that children exposed to fly ash exhibit significantly higher depressed symptoms, with a Depressive Problems Scale (DSM) score of 3.13 points (95% CI = 0.39, 5.88) higher than children who are not exposed^12^. Our study extends this insight further by focusing on the direct association between fly ash exposure and depressive symptoms.

The current study aims to fill these gaps by examining the mental health effects of fly ash exposure, specifically focusing on individuals with different BMI categories (overweight vs. normal). We hypothesized that those who are overweight and exposed to fly ash may be at higher risk for psychological disorders. Additionally, we aim to develop a predictive machine learning model to identify individuals at greater risk for mental health issues due to environmental factors.

The research aims to achieve the following key objectives: (i) Assess psychological disorders (depression, anxiety and stress) among the population living near coal- thermal power plant; (ii) examine the association between psychological disorders and factors such as age, gender, household air pollution, and socioeconomic status; (iii) investigate the relationship between depression and factors like stress, anxiety, fuel type, age group and income, while developing a predictive model for depression and finally (iv) compare mental health outcomes between overweight and normal-weight individuals living near coal thermal power plant.

The results of this study would provide valuable insight into the effects of coal ash on mental health and help address a significant research gap regarding environmental pollutants. The findings could inform public health policymakers to promote stricter environmental regulations and support communities in advocating for improved living conditions, ultimately leading to better mental health outcomes for affected communities.

## Methods

### Selection of a thermal power plant

Guru Gobind Singh Super Thermal Power Plant (GGSSTPP), located in Rupnagar, is one of the oldest and largest operational thermal power plants in Punjab, which was commissioned in the 1980s. Its long history of operation has led to sustained and cumulative emissions, making it an ideal site to study the long-term exposure effects on nearby residents. The densely populated villages within a 5 km radius of GGSSTPP enabled us to enroll a large, stable, and diverse group of study participants. These villages experience limited migration, ensuring consistent environmental exposure among the residents. Furthermore, government and media reports have highlighted ongoing community complaintsregarding air quality and health concerns in this region, emphasizing the relevance and urgency of this investigation.

### Study population

This cross-sectional study was conducted from October 2018 to March 2019 around the GGSSTPP, in Rupnagar, Punjab, India. The target population consisted of residents from villages located between 1 and 5.5 km from the power plant. The selected distance range was based on existing literature and environmental health guidelines, which suggest that populations within a 5 km radius of coal thermal power plant are at higher risk of exposure to air pollutants and associated health risks^13,14^ The lower limit of 1 km was set to exclude areas within the immediate industrial zone, where population density is low and direct occupational exposure might confound community-level findings. The upper limit was slightly extended to 5.5 km to ensure adequate sample size and to capture populations on the fringe of the commonly studied 5 km zone, allowing for a more robust assessment of spatial exposure gradients. Data was collected from the following villages: Alipur, Ghanouli, Kotbala, Majri, Begumpura, Saini Majra, Jatt Patti, Rattanpura, Singhpura, Inderpura, Rawal Majra, and Doburji. The study included individuals aged 19–60 who had lived near the area for more than 5 years.

### Exclusion criteria

Pregnant women and adults who had relocated within the past 2 years were excluded. Long-term exposure to environmental stressors frequently results in mental health issues such as stress, anxiety, and depression. Individuals who had only lived there for a short time would have introduced variability in exposure levels, potentially complicating the results. To strengthen the validity of its findings, the study focused on long-term residents, ensuring consistent and significant exposure histoies among participants.

### Survey design

A two-stage random sampling was used for data collection, with the Rupnagar district as the unit of analysis. The first stage involved selecting villages, followed by a random selection of households within those villages. The sample area was determined using municipal wards, census blocks, or community development blocks as units. Field investigators and medical social workers addressed the village sarpanch (head) or other community leaders to solicit their aid in establishing the survey’s boundaries and encourage community participation.

Using quota sampling, ensuring adequate representation of the population across age groups, genders, and socioeconomic statuses, reflecting the demographic diversity of the area^15^. We selected the communities within 5 kilometers of the power plant to capture data from different exposure levels. This approach aligns with standard practices in environmental health research for assessing pollution impacts^16^. By using villages/colonies as the primary unit and households as the secondary unit, this method minimizes selection bias and improves representativeness^17^.

### Sample size

To get the representative sample size, we applied the following formula

$$n\,=\,\left({Z}^{2}* p\left(1-p\right)\right)/\,{{\rm{e}}}^{2}$$

where

- *n* = sample size,
- *z* = *z* – score of the selected level of confidence,
- *p* = standard deviation (use 5 if unknown depending on the study)
- *e* = margin of error
- For a 95% confidence level and 5% margin of error, which are commonly used in research, the calculation would be

$${n}=({1.96}^{2}* 0.5(1-0.5))/0.0{5}^{2}=384.16$$

The final sample size for our study was 359 participants from 12 villages within a 5.5 km radius of the coal thermal power plant, slightly below the typical sample size of 385. This sample size was chosen to ensure population representativeness and sufficient statistical power. A sample size of 359 is nearly equivalent to the sample size of 385 for most population sizes, providing sufficient statistical power to ascertain distinguishing sample differences^18^. To account for a 10% anticipated non-response rate, adjustments were incorporated into sample size estimation. This approach ensures that analyses have the statistical power necessary to identify significant impacts.

### Socioeconomic stratification

To explore the relationship between economic disparities, environmental risks, and mental health, participants were divided into two income groups: those earning less than one lakh (~1200$) and those earning more than one lakh^19^.

### Household air pollution assessment

Based on known associations between solid fuel use and health risks in poor countries, participants were categorized based on the type of fuel used in their household: solid/mixed fuels vs. clean fuels—LPG^20^. This multimodal sampling aims to ensure that the sample accurately reflects the diverse population affected by pollution from coal thermal power plants. It enables broader applicability of results and a comprehensive understanding of how environmental and socioeconomic factors influence psychological symptoms in populations affected by pollution.

### Study questionnaire

The study area was divided into buffer zones ranging from 1 to 2 km around the centroid of the power plant. The Depression, anxiety and stress scale (DASS-21) questionnaire was used to assess psychological symptoms. The DASS-21 is a reliable and user-friendly tool, widely used in both clinical and research settings. It includes 21 self-rated questions covering three scales: depression, anxiety, and stress, with 7 items per scale. The depression scale assesses feelings of hopelessness, self-deprecation, and anhedonia, while the anxiety scale measures autonomic arousal and anxious affect. The stress scale evaluates chronic nonspecific arousal, including nervousness and irritability^18^.

Participants were categorized based on age, gender, income, and cooking fuel types (LPG or mixed fuels). A total of 359 individuals were included in the study, divided into normal BMI and overweight BMI^21^. Of the participants, 145 had normal BMI, while 214 were classified as overweight BMI. Psychological symptoms (depression, anxiety, stress) were further categorized based on DASS-21 guidelines into two groups: those with psychological symptoms and those without, after adding all the scores.

### Statistical analysis

Data analysis was conducted using binary logistic regression to examine the association between psychological symptoms and factors such as household air pollution, income, age, and gender. Odds ratios were calculated to assess the impact of each factor on psychological symptoms. For BMI, participants were categorized into two groups: normal and overweight, based on established criteria^22^. Age was divided into three groups: 19–29 years, 30–49 years, and 50+ years^15^. Income was classified into two categories: less than one lakh and one lakh or more^19^. Gender was categorized as male or female, and household air pollution was classified into two categories: solid/mixed fuels and clean fuels (LPG). For the depression predictive model, age and income were treated as continuous variables, with the goal of assessing how changes in age or income affect the odds of depression using binary logistic regression.

A total of 359 participants were included to develop a predictive model for depression using binary logistic regression. Binary logistic regression is used when the dependent variables are binary (e.g., Yes vs. No), with independent variables that can be categorical, continuous, or both^23^^,^^24^ The analysis was performed using SPSS Statistics 26 and R Studio v4.2.2.

Additionally, the association between depression and independent variables (age, gender, cooking fuel types, income, stress, and anxiety symptoms) in the general population was analyzed using binary logistic regression. A depression predictive model was also developed in R Studio v4.2.2. To compare normal and overweight participants, statistical differences were assessed using *t*-tests and ANOVA tests in the SciPy library of Python.

### Ethics approval

The study was approved by the Institutional Ethics Committee, PGIMER, Chandigarh vide letter No. PGI/IEC/2017/97.

### Consent to participate

Participants were fully informed about the study’s purpose and procedures. They signed a consent form indicating their voluntary participation and acknowledging that all information provided would be kept confidential.

### Assumptions

The applicability of our findings to a larger population is limited by the small sample size of 359 individuals, which may affect subgroup analyses, such as age or gender differences. Small sample sizes can lead to type II errors, obscuring important correlations^25^^,26^. Additionally, the cross-sectional design makes it challenging to establish causality between environmental factors and mental health outcomes. While correlations between fuel consumption and mental health symptoms were observed, we cannot determine the directionality of these relationships or rule out confounding factors, such as pre-existing mental health conditions. As highlighted in a previous study^23^ longitudinal studies would be better suited for exploring these causal links.

Gender-related biases may also influence our findings, as females are more likely to report psychological issues compared to males^27^. Furthermore, our study was limited to neighborhoods near a single power plant; this might affect the generalizability, as results may not apply to areas with different pollution levels or socioeconomic conditions. Environmental health impacts can vary widely across regions^28^. Finally, while we used fuel type as a proxy for pollution exposure, we did not measure individual-level exposure. This limitation may have led to less precise estimates of the relationship between pollution and mental health outcomes. Hence, as mentioned by Reuben et al.^29^ more accurate exposure assessment techniques could provide deeper insights into this connection. We had also considered assumptions like there should not be multicollinearity in the datasets and linearity in the logit.

Another limitation of this study is the time gap in data collection (2018–2019). While sociodemographic and environmental variables in the region have largely remained unchanged, there may have been shifts in healthcare access, environmental factors, or public health policies that could affect the applicability of our findings to the current situation. However, we believe the findings still highlight the ongoing mental health in communities near thermal power plants, especially in the absence of major infrastructure improvements or interventions.

## Results

### Socio-demographic analysis

Participant's age ranged from 19 to 71 years, with a mean of 43.79 and a median of 44. Income levels varied from 0.15 to 8 lakhs, with an average income of 1.68 lakhs. The age, as well as the income distribution, indicates moderate diversity regarding the socioeconomic and demographic background of the samples. Among the residents near power plants, 238 used LPG, while 121 used mixed fuel types, as shown in Table 1. The sample consisted of 29% male and 70% female participants. Out of the 359 participants, 45 (12%) showed symptoms of depression, 78 (21.7%) had anxiety, and 20 (5%) expressed stress. Females were more likely to exhibit symptoms of depression, anxiety, and stress. Specifically, 73.44% of those with depression, 79.48% with anxiety, and 55% of those with stress were female, as shown in Table 2. Analysis of gender-stratified prevalence of mental health conditions revealed distinct patterns. Among females (*n* = 253), depression was observed in 13.04% of participants compared to 11.32% in males (*n* = 106), yielding an overall female prevalence of 9.19% (33/359) versus 3.34% (12/359) in males. Anxiety demonstrated a more pronounced gender disparity, with 24.50% of females experiencing symptoms (62/253; 17.27% of total population) compared to 15.09% of males (16/106; 4.45% of total population). Stress prevalence exhibited a contrasting pattern, with 4.34% of females (11/253; 3.06% of the total population) reporting symptoms versus 8.49% of males (9/106; 2.50% of the total population). These findings indicate that while depression and anxiety demonstrate higher prevalence among females, stress symptoms were more frequently reported by male participants, but in the overall population, females are more stressed.

**Table 1:** Demographic characteristics of the study participants

Variable	Count	Percentage (%)
*Age group*		
19–29 Years	43	11.98
30–49 Years	196	54.60
≥50 Years	120	33.43
*Gender*		
Male	106	29.53
Female	253	70.47
*Fuel*		
LPG	238	66.3
Wood	121	33.7
*Income group*		
<1 Lakh	156	43.45
≥1 Lakh	203	56.55
*Depression status*		
Depressed	45	12.53
Normal	314	87.47
Depressed female%	33	9.19
Depressed male%	12	3.34
*Anxiety status*		
Normal	281	78.27
Anxious	78	21.73
Anxious in female%	62	17.27
Anxious in male%	16	4.45
*Stress status*		
Normal	339	94.43
Stress-prone	20	5.57
Stress-prone-female%	11	3.06
Stress-prone-male%	9	2.50
*BMI*	Mean ± SD = 26.59 ± 5.37	

The table presents the demographic characteristics of the study participants, with three columns specifying the variables (age, gender, types of cooking fuel, income group, depression status, anxiety status, stress status, and BMI), the number of counts, and the percentage of each group/category. The BMI of the participant is represented as the mean ± standard deviation.

**Table 2:** Frequency distribution, association, and odds ratio of depression, anxiety, and stress by age groups, gender, cooking fuel type, and income groups in overweight (O) and normal (N) BMI population

		Age			Gender		Types of fuel		Income	
		19–29 Years	30–49 Years	≥50 Years	Male	Female	LPG	Mixed	<1 lakh	≥1 Lakhs
*Depression*	No(O)	8	111	70	46	143	131	58	35	154
	No(N)	30	61	34	48	77	76	49	29	96
	Yes(O)	2	13	10	7	18	19	6	3	22
	Yes(N)	3	11	6	5	15	12	8	3	17
	% Yes(O)	20%	10.48%	12.5%	13.2%	11.18%	12.66%	9.37%	7.89%	12.5%
	% Yes(N)	9.09	15.28	15.00	9.43	16.30	13.64	14.04	9.38	15.04
	*P*-Value(O)	0.439	0.810	–	–	0.790	–	0.491	0.412	–
	*P*-Value(N)	0.626	0.962	–	–	0.325	–	0.893	0.469	–
	Odd Ratio(O)	1.965	0.896	1	1	0.879	1	0.710	0.583	1
	Odd Ratio(N)	0.685	0.973	1	1	1.760	1	1.071	0.609	1
*Anxiety*	No(O)	8	94	69	47	124	117	54	28	143
	No(N)	25	53	32	43	67	69	41	69	41
	Yes(O)	2	30	11	6	37	33	10	10	33
	Yes(N)	8	19	8	10	25	19	16	19	16
	% Yes(O)	20.00	24.19	13.75	11.32	22.98	22.00	15.63	26.32	18.75
	% Yes(N)	24.24	26.39	20.00	18.87	27.17	21.59	28.07	21.59	28.07
	*P*-Value(O)	0.634	0.121	–	–	0.081	–	0.226	0.442	–
	*P*-Value(N)	0.276	0.423	1	1	0.257	1	0.190	0.045	1
	Odd Ratio(O)	1.521	1.847	1	1	2.306	1	0.614	1.391	1
	Odd Ratio(N)	1.921	1.490	1	1	1.677	1	1.721	0.303	1
*Stress*	No(O)	9	118	75	49	153	139	63	36	166
	No(N)	30	69	38	48	89	87	50	31	106
	Yes(O)	1	6	5	4	8	11	1	2	10
	Yes(N)	3	3	2	5	3	1	7	1	7
	% Yes(O)	10%	4.83%	6.25%	7.54%	4.96%	7.33%	1.56%	5.26%	5.68%
	% Yes(N)	9.09	4.17	5.00	9.43	3.26	1.14	12.28	3.13	6.19
	*P*-Value(O)	0.596	0.801	–	–	0.577	–	0.132	0.873	–
	*P*-Value(N)	0.289	0.956	–	–	0.314	–	0.011	0.226	–
	Odd Ratio(O)	1.883	0.850	1	1	0.697	1	0.203	0.875	1
	Odd Ratio(N)	3.126	0.947	1	1	0.427	1	17.408	0.244	1

This table provides the frequency distribution of depression, anxiety, and stress in overweight and normal BMI Individuals. The normal BMI (N) and Overweight (O) populations are classified into two categories: (a) Symptoms present (Yes) and (b) Symptoms absent (No). The cross-sectional distribution is further explained by various independent parameters including age, gender, types of fuel, and income groups. The association between depression, anxiety, and stress in individuals with overweight and normal BMI and the independent parameters are expressed in terms of odd ratios and *p*-values. A *p*-value < 0.05 indicates a statistically significant association, while a *p*-value ≥ 0.05 indicates no significant association.

### Depression among overweight people

It was noticed that depression was more prevalent among overweight males (13.2%) than females (11.18%), suggesting higher rates of depression in males. Previous studies indicated that depression symptoms tend to decrease with a lower BMI^30^. The highest depression rates were observed in the 19–29 age groups, with about 20% affected.

**Fig. 1:**
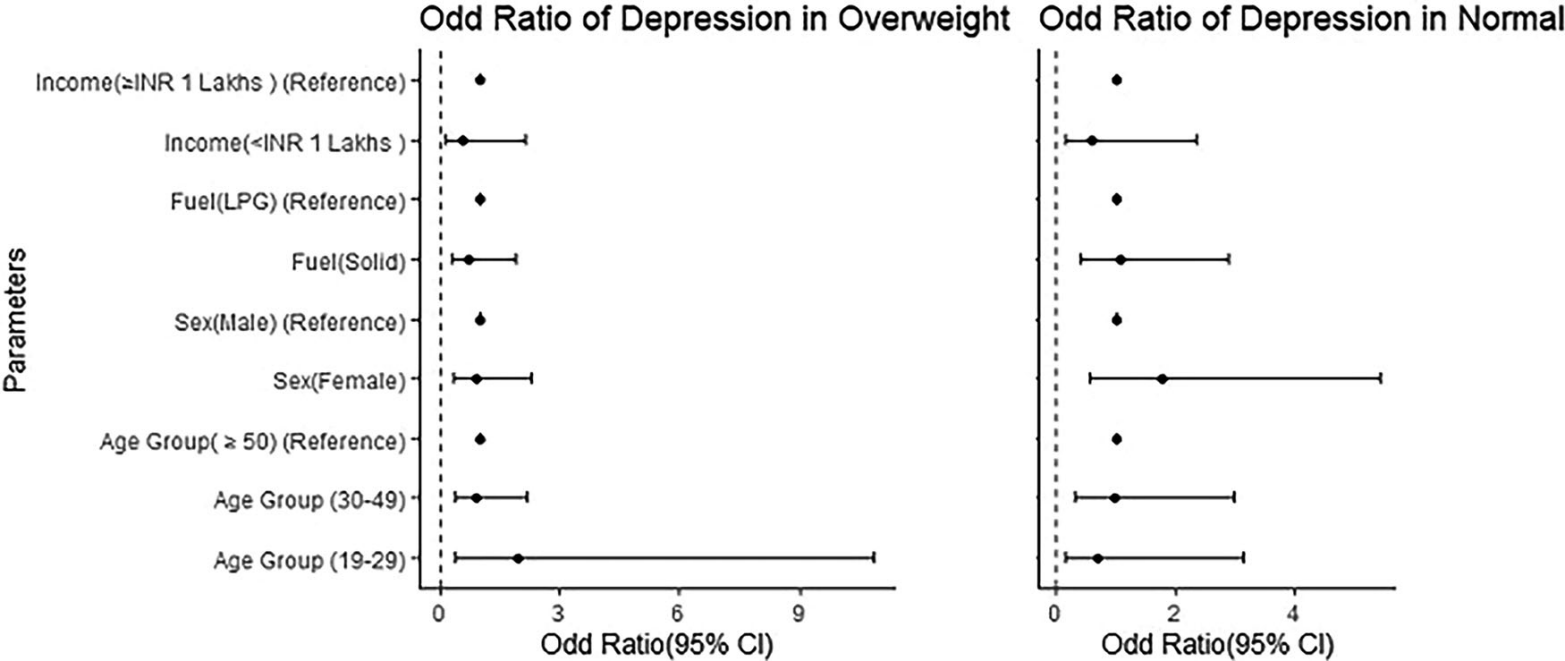
Odd Ratio (95% CI) forest plot of depression with age group, sex, cooking fuel types, and income group in overweight and normal BMI population. The left panel shows the odds ratio for depression in individual overweight BMI, while the right panel displays the odds ratio for depression in individuals with normal BMI. The *y*-axis represents different parameters, and the *x*-axis shows the odds ratio with a 95% confidence interval. The bold dot indicates the exact odds ratio, and the horizontal line extending from the dot represents the 95% CI values. The reference odd ratio value is set at 1.

Additionally, individuals with an income below 1 lakh were more prone to depression than those with higher incomes. Previous studies have linked depression to obesity^31^ and found a connection between depression, overweight, and obesity in adolescents. However, our study found no significant association between depression and age, cooking fuel type, gender, or income (*p*-value ≥ 0.05). The odds ratio for depression in the 19–29 and 30–49 age groups compared to those aged 50 above was 1.965 (95% CI, 0.355–10.883) and 0.896 (95% CI, 0.366–2.194), respectively. Previous studies have suggested a link between depression and household air pollution^28^^,32^. It was also found that the odds ratio for depression was higher in males and individuals with incomes above one compared to those earning less (Fig. 1).

### Depression among normal people

The analysis revealed that depression in individuals with normal BMI was not significantly associated with cooking fuel type, income, gender, or age group. Among this group, the highest proportion of depression symptoms (15.28%) was observed in the 30–49 age group, followed by 15% in those aged 50 above and 9.09% in the 19–29 age group. Females (16.30%) showed a higher proportion of depression symptoms than males (9.43%). Additionally, individuals using mixed had higher depression rates (14.04%) compared to those using LPG (13.64%). People with an income above 1 lakh also showed a higher depression rate (15.04%) compared to those with an income below 1 lakh (9.38%), as shown in Table 2.

### Anxiety among overweight people

**Fig. 2:**
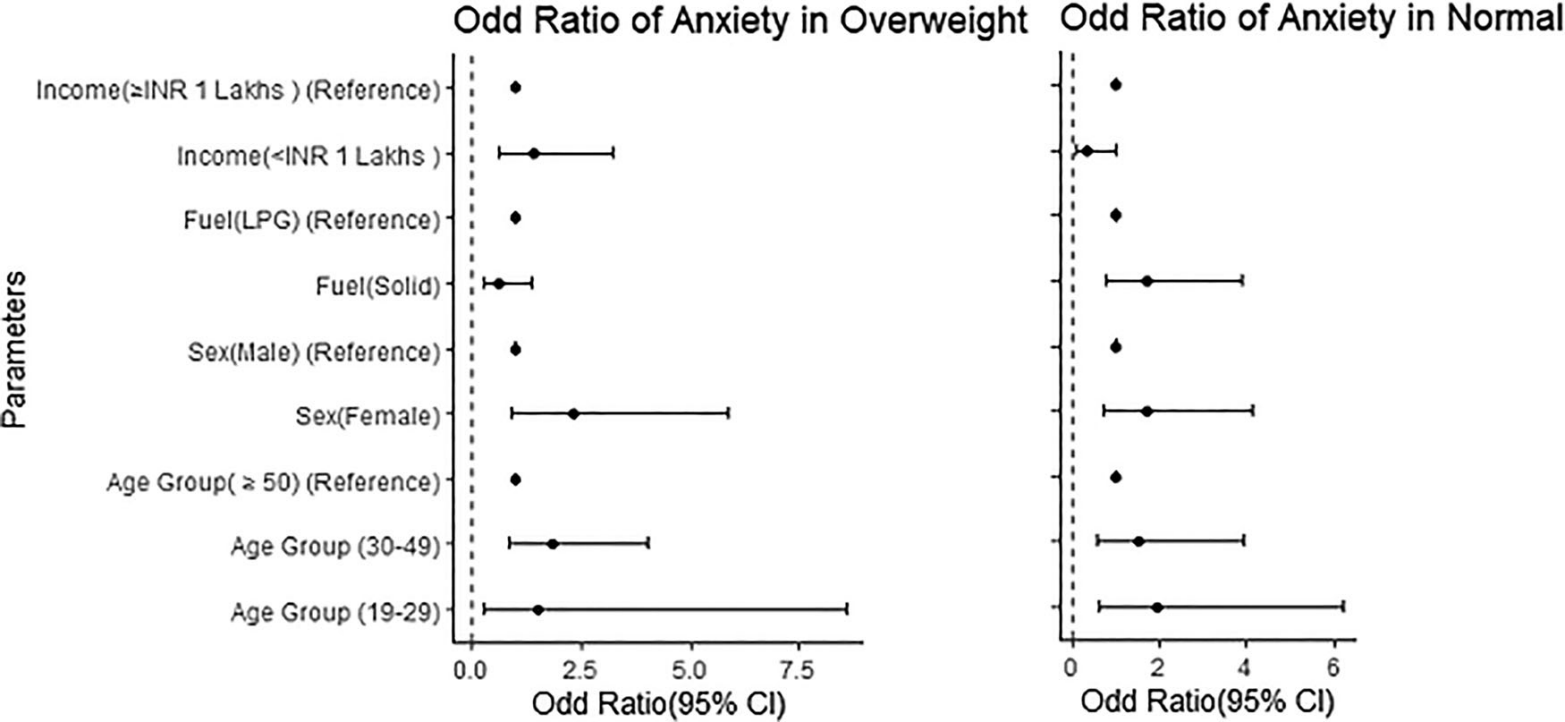
Odd Ratio (95% CI) forest plot of anxiety by age group, sex, cooking fuel types, and income group in overweight and normal-weight populations. The left panel shows the odds ratio of anxiety in the overweight population, while the right panel displays the odds ratio for anxiety in individuals with normal BMI. The *y*-axis represents different parameters, and the x-axis shows the odds ratio with the 95% confidence interval of anxiety. The bold dot indicates the exact odds ratio, and the horizontal line extending from the dots represents the 95% CI. The reference odd ratio value is set at 1.

Anxiety was found to be more prevalent among overweight females (22.98%) compared to males (11.32%). The highest anxiety symptoms were found in the 30–49 age group (24.19%). Additionally, anxiety is more common in those using LPG compared to mixed fuels. People with an annual income below 1 lakh were found to be more vulnerable to anxiety than those with higher incomes. However, anxiety is not significantly associated with age group, fuel type, gender, or income. The odd ratios for anxiety in the 19–29 and 30–49 age groups compared to those over 50 years are years is 1.521 (95% CI, 0.270–8.567) and 1.84 (95% CI, 0.903–5.889), respectively. The odds of anxiety were higher in females, LPG users, and those with an income below 1 lakh, as shown in Fig. 2.

### Anxiety among normal-weight people

The study found a significant association between anxiety and income below 1 lakh in normal BMI individuals (*p* = 0.045, OR = 0.303, 95% CI), the odds ratio of 0.303 for anxiety suggests that normal BMI individuals with income below 1 lakh had approximately 70% lower odds of experiencing anxiety compared to their higher-income counterparts. Anxiety symptoms were more prevalent in the 30–49 age group (26.39%), followed by the 19–29 age group (24.24%) and those aged 50 and above (20%). Anxiety was also more common in females (27.17%) than in males (18.87%), consistent with previous studies^31^. Additionally, anxiety was more frequent among individuals exposed to household air pollution (28.07%) compared to those using mixed fuel (21.59%). Those with an income above 1 lakh also experienced anxiety (28.07%) compared to those with an income below 1 lakh (21.59%). The odds ratio for anxiety was higher in females, those using mixed fuel, and those with incomes of 1 lakh or more, as shown in Fig. 2.

### Stress among overweight people

**Fig. 3:**
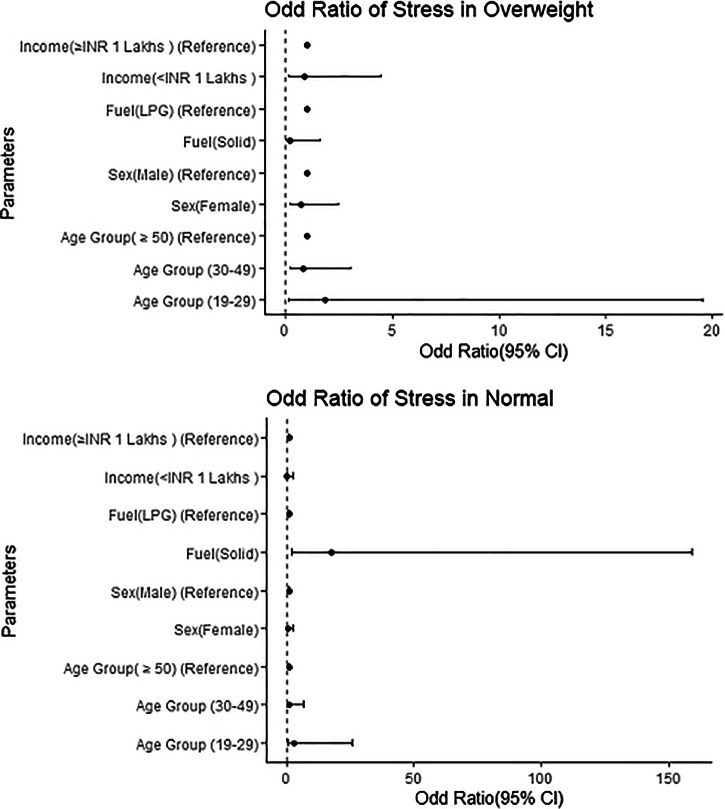
Odd Ratio (95% CI) forest plot of stress by age, sex, cooking fuel types, and income group in overweight and normal-weight populations. The *y*-axis represents different independent parameters, and the *x*-axis shows the odds ratio with the 95% confidence interval for stress. The bold dot indicates the exact odd ratio, and the horizontal line extending from the dot represents the 95% CI. The reference odd ratio value is set at 1.

It has been noticed that stress in overweight individuals is more prevalent in males, 7.54%, than in females (4.96%). The highest stress levels were observed in the 19–29 age group (10%). Stress symptoms were also found to be more common in those using LPG compared to those using mixed fuels. Additionally, individuals with an income above 1 lakh were found to be more prone to stress than those earning less than 1 lakh. However, stress does not appear to be significantly associated with age, fuel type, gender, or income. The odds ratio for stress in the 19–29 and 30–49 age groups compared to those over 50 years is 1.883 (95% CI, 0.182–19.532) and 0.85 (95% CI, 0.239–3.017), respectively. In overweight individuals, the odds ratio of stress are higher in males, LPG users, and those with an income above 1 lakh, as depicted in Fig. 3.

### Stress among normal people

The study found a significant association between stress and household air pollution (*p* = 0.011, OR = 17.408, 95% CI), with an odds ratio of 17.408 indicating that individuals exposed to household air pollution were approximately 17 times more likely to experience stress symptoms. The highest stress levels were found in the 19–29 age group (9.09%), compared to those above 50 and above (5%) and 30–49 years (4.17%). Stress was found to be more common in males (9.43%) than in females (3.26%). Individuals using solid biomass fuels (12.28%) reported more stress than those using clean fuel (1.14%). Additionally, stress is more prevalent in those with an income above 1 lakh compared to those earning below 1 lakh. In normal BMI individuals, the odd ratios of stress were found to be higher in males, those using mixed fuels, those with an income of 1 lakh or more, and those in the 19–29 age groups, compared to females, LPG users, those with an income below 1 lakh, and the 50 above age group (compared to 30–49 years), as shown in Fig. 3. These findings were found to be aligned with previous studies^33^^,34^.

### Depression in the general population and the development of a depression predictive model

**Fig. 4:**
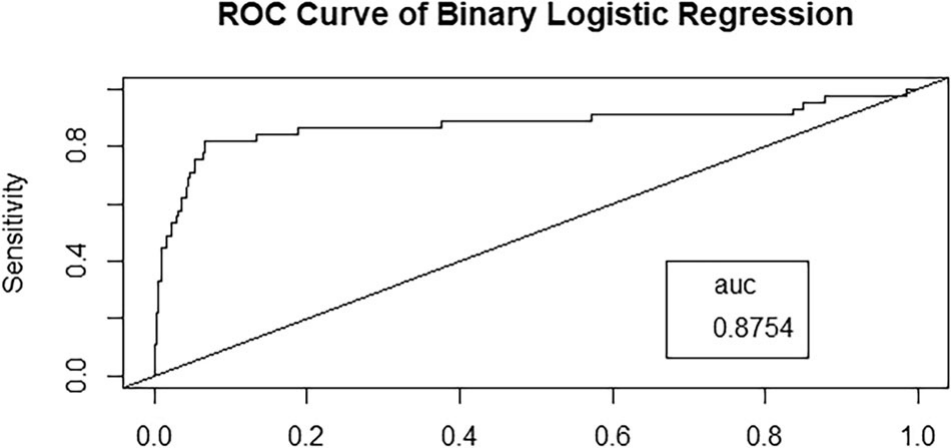
Receiver operating characteristics–area under the curve (RoC–AUC) of the binary logistic regression model for depression prediction. The *y*-axis represents the sensitivity of the model (true positive rate), while the *x*-axis shows the false positive rate. The curve illustrates the model’s performance, plotting the true positive rate against the false positive rate. The diagonal line represents a random classifier. The RoC–AUC curve ranges from 0 to 1, with the area under the curve for this model being 0.875.

This study found a significant association between depression and age (Odd ratio-1.05, *p*-value-0.011), anxiety (Odd ratio-28.07, *p*-value-0), and stress (Odd ratio-11.10, *p*-value-0). A binary logistic regression model was used to examine the relationship between depression and age, anxiety, stress, income, and cooking fuel types, as shown in Table 3. The depression predictive model demonstrated an ROC–AUC of 0.8754, indicating good predictive accuracy, as illustrated in Fig. 4.

**Table 3:** Association of depression with different parameters using binomial logistic regression (age, gender, fuel type, income, anxiety, and stress)

Parameters	Age (continuous) (*)	Gender (female wrt male)	Fuel (mixed wrt LPG)	Income (Continuous)	Anxiety (Yes wrt No) (***)	Stress (Yes wrt No) (***)
*P*-value	0.011	0.732	0.545	0.696	0	0
Odd-Ratio	1.053	0.842	0.759	0.930	28.07	11.10

This table presents the association of depression with various eco-socioeconomic parameters, using binomial logistic regression. The significant codes are as follows: ***p < 0.001, **p < 0.01, *p < 0.05, *p* < 0.1, and no symbol *p* > 0.05. The results indicate that depression is statistically significantly associated with age, stress, and anxiety.

### Statistical difference between overweight and normal-weight individuals

ANOVA and *t*-tests were used to compare overweight and normal-weight populations, as shown in Table 4 and Table 5. The analysis revealed significant in age and gender between the two groups. Age was significantly different, as estimated by both the *t*-test (*t*-statistic = 4.6693, *p*-value < 0.05) and ANOVA (*F*-statistic = 24.1235, *p*-value < 0.05), indicating that the overweight group is significantly older, as also depicted in Table 3 and Table 4. Gender distribution also showed significant differences, with both the *t*-test (*p*-value < 0.05) and ANOVA (*p*-value < 0.05) suggesting variation between the two groups. The type of cooking fuels approached significance (*t*-test *p*-value = 0.09483, ANOVA *p* = 0.09107) but did not meet the threshold for significance. Other variables, including income, depression, anxiety, and stress, showed no significant differences (*p*-values > 0.05), indicating no substantial variation between the groups.

**Table 4:** ANOVA test comparing the difference between normal and overweight Individuals

Parameters	*f*-statistics	*p*-values
Age	24.123	<0.05
Gender	5.559	<0.05
Cooking fuel	2.871	>0.05
Annual income	0.159	>0.05
Depression	0.562	>0.05
Anxiety	1.518	>0.05
Stress	0.022	>0.05

This table presents the results of an ANOVA test comparing the means between two groups: normal BMI and overweight individuals. The *f*-statistics represents the ratio of the variance between groups to the variance within the groups. The *p*-value indicates the statistical significance of the results, with the lower *p*-values suggesting a more significant difference between the two groups.

**Table 5:** *t*-Test comparing the difference between normal and overweight Individuals

Parameters	*t*-statistic	*p*-values
Age	4.6693	<0.05
Gender	2.3107	<0.05
Cooking fuel	−1.6758	>0.05
Annual income	−0.3841	>0.05
Depression	−0.7481	>0.05
Anxiety	−1.2353	>0.05
Stress	0.1516	>0.05

This table presents the results of a two-sample *t*-test comparing the means between normal BMI and overweight participants. The *t*-statistic indicates whether the means of the two groups are significantly different, considering the variability within each group and the sample size. The *p*-value shows the statistical significance of the results, with a lower *p*-value indicating a significant difference between the groups.

## Discussion

This study has important implications for public health and policy, particularly in understanding the relationships between socioeconomic factors, environmental influences, and mental health. Our findings provide several key insights:

We found a strong link between elevated levels of stress, anxiety, depression, and household air pollution. This is consistent with previous studies showing the impact of environmental factors on mental health^32^. Recent reviews have further confirmed that air pollution is associated with increased risk of various psychiatric disorders, including depression, schizophrenia, and bipolar disorder, with neuroinflammatory mechanisms potentially underlying these associations^35^. These results highlight the need for targeted interventions to reduce pollution exposure in at-risk groups, particularly those using solid biomass fuels, a known risk factor for mental health issues^20^. Further, the study shows that females are more likely to experience anxiety and depression than males at all BMI and income levels.

This gender gap aligns with other studies^27^ suggesting that females may be more vulnerable to the effects of environmental pollution or face specific psychosocial pressures. Evidence from international studies indicates that the cognitive and mental health effects of air pollution are often pronounced in females than in males, particularly in non-metropolitan areas, highlighting the need for gender-sensitive interventions^36^. The higher prevalence of depression, anxiety, and stress in females corresponds with Zierold et al.,^12^ who found increased vulnerability to environmental pollutants in certain demographic groups^12^. These findings emphasize the importance of gender-sensitive public health policies to address the mental health needs of females in polluted areas.

Additionally, our study emphasizes the importance of addressing broader social determinants of mental health, such as income inequality and access to mental health services, alongside promoting cleaner fuels^37^. Individuals with normal BMI with incomes more than ₹1 lakh were 70% more likely to suffer from anxiety as compared to incomes below ₹1 lakh, underscoring the significant role of economic factors in mental health and suggesting that policies should address these. Income-based inequities in access to mental health services have been documented globally with lower-income individuals. We need medical attention for higher-income groups as well^38^. The findings have significant implications for environmental health initiatives, such as the PMUY. These programs should integrate mental health components, including assessments and support for communities transitioning to cleaner fuels. Efforts to reduce household air pollution, given its substantial impact on mental health^39^.

Finally, our study calls for focused mental health support for those affected by environmental contamination. Public health strategies should be comprehensive, addressing both psychological and physical health impacts. This could include community-based mental health programs, mental health screenings, and public awareness campaigns on the effects of environmental pollution on mental health^8^. Recent research has shown that children and adolescents are particularly vulnerable to coal ash and other pollutant exposure. exhibits higher rates of depression and neurodevelopmental issues^12,37^. These findings highlight the need for integrated public health strategies that address both socioeconomic and environmental factors influencing mental health in at-risk groups. The significant association between stress and household air pollution aligns with emerging evidence that air pollutants can trigger psychological responses^40^. Moreover, the interaction between social and environmental factors is complex and dynamic. Factors such as social support and access to green spaces can mitigate the mental health effects of environmental stressors^41,42^. Interventions that enhance resilience, such as strengthening community networks or improving urban environments, may help buffer the psychological impact of pollution.

Our findings highlight key areas for further research to better understand the complex relationships between socioeconomic factors, environmental degradation, and mental health. First, longitudinal studies are essential to establish a causal link between environmental pollution and mental health. While our cross-sectional study offers valuable insights, tracking individuals over time would help determine whether pollution exposure directly causes stress, anxiety, or depression^29^. Such studies could clarify the temporal dynamics and cumulative effects of prolonged exposure. Longitudinal and spatial-temporal studies have shown that living in more polluted areas is associated with poorer mental well-being, especially among ethnic minorities and non-native populations. This highlights the need for geographically and culturally tailored interventions^43^.

Second, future research should involve larger and more diverse participant groups to improve generalizability. Expanding the study beyond our geographic region to include different locales, socioeconomic backgrounds, and exposure levels would help determine whether the observed relationships are context-specific or universally applicable^44^. This approach could also reveal cultural or regional differences in how pollution impacts mental health.

Third, future studies should more thoroughly control for confounding variables, such as social support, employment status, and past mental health history, and also should explore specific biological mechanisms linking power plant emissions to mental health outcomes and evaluate targeted interventions for at-risk populations, particularly women and low-income households Kumar et al.^45^. This would enable a more accurate assessment of the individual effects of environmental pollution and socioeconomic factors on mental health outcomes^37^. Additionally, future research should explore whether financial stress related to fuel prices or challenges in transitioning to cleaner fuels contributes to mental health issues^46^. Building on our study, addressing these directions will enhance our understanding of how socioeconomic and environmental factors interact to affect mental health and inform more targeted public health interventions and policies.

The study revealed that females experience higher levels of depression, anxiety, and stress compared to males. For the overweight population, no significant association was found between psychological symptoms and demographic factors such as age, gender, income, or household air pollution. In contrast, for individuals with a normal BMI, anxiety was 70% lower odds of experiencing anxiety compared to their higher-income counterparts, while stress was correlated with household air pollution. Additionally, depression was notably associated with age and also linked to anxiety and stress levels.

These findings emphasize the need for public health policies that address mental health issues, particularly for female populations. Future environmental health programs should include psychological components to address the mental health effects of exposure to coal power pollution. However, the generalizability of our findings is limited by the small sample size and the focus on a single geographical location without direct measurement of ambient environmental factors, which could influence the observed mental health outcomes. Future research should include larger, more diverse samples and better pollution exposure assessments to enhance real-time monitoring of meteorological variables and the applicability of the findings. Expanding studies to different regions with varied pollution levels and socioeconomic backgrounds will help inform more effective public health policies.

## Data Availability

Data will be available on request.

## Code availability

Code will be available on a knowledge-sharing basis, as it includes intellectual content.
